# The Application of ^99m^Tc-DTPA Renal Dynamic Imaging to Measuring Renal Function of Children with Acute Lymphoblastic Leukemia after Induction Therapy

**DOI:** 10.1155/2020/3687134

**Published:** 2020-11-17

**Authors:** Lidan Wang, Kailan Chen, Qiong Xu

**Affiliations:** ^1^Department of Radiology, Wuhan Children's Hospital, Tongji Medical College, Huazhong University of Science & Technology, China; ^2^Department of Hematology and Oncology, Wuhan Children's Hospital, Tongji Medical College, Huazhong University of Science & Technology, Wuhan, Hubei, China; ^3^Department of Clinical Pharmacology, Wuhan Children's Hospital, Tongji Medical College, Huazhong University of Science & Technology, Wuhan, Hubei, China

## Abstract

**Purpose:**

The study was aimed at assessing renal functions of children with acute lymphoblastic leukemia (ALL) after induction therapy by ^99m^Tc-DTPA renal dynamic imaging Gates method (GFR_Gates_) and investigating whether renal function after induction therapy will affect the occurrence of high-dose methotrexate- (HDMTX-) induced acute kidney injury (AKI).

**Methods:**

Children with newly diagnosed ALL were enrolled. Renal functions before the administration of HDMTX were assessed by estimated glomerular filtration rate (eGFR) and GFR_Gates_, respectively, before the first cycle of HDMTX after induction therapy. The areas under the ROC curve were used to assess covariates' ability to predict HDMTX-induced AKI.

**Results:**

102 children with ALL were included in the study. A stepwise backward binary logistic regression showed that only standardized GFR_Gates_ was an independent risk factor for HDMTX-induced AKI (*p* = 0.018, odds ratio 0.985, 95% CI 0.972-0.997). The area under the ROC of standardized GFR_Gates_ was 0.679 (*p* = 0.012, 95% CI 0.554-0.804).

**Conclusion:**

Standardized GFR_Gates_ showed that the normal renal function of children is not enough to be used as a cutoff point to predict HDMTX-induced AKI in ALL children receiving HDMTX. More attention and supportive care should be given to the children with standardized GFR_Gates_ lower than the cutoff value to avoid the HDMTX-induced AKI.

## 1. Introduction

Extramedullary infiltration is a significant cause of treatment failure of acute lymphoblastic leukemia (ALL) after complete molecular remission. High-dose methotrexate (HDMTX) therapy combined with intensified intrathecal chemotherapy has been proved to be effective in decreasing the central nervous system relapse of ALL [1]. Now, HDMTX is an important component of intensive systemic chemotherapy.

Keeping serum MTX concentrations within the target concentration range is important as it can decrease early relapse and prevent dose-related side effects. Research showed that there was a correlation between serum MTX concentration and treatment effects [2, 3]. The risk of relapse was lower in patients who had serum MTX concentrations within the target range. On the other hand, MTX concentrations were found to be correlated with toxicity [4], and overdosing might be life-threatening [5]. Nephrotoxicity is especially troublesome as it can delay the MTX excretion, prolong exposure to toxic MTX concentrations, and exacerbate other toxicities [6, 7].

Many factors were reported to affect the MTX concentrations [8]. The administration of HDMTX was deeply studied in patients with risk factors of methotrexate toxicities. Glomerular filtration function (GFR) is an important factor that could influence MTX clearance in patients receiving HDMTX [9]. In patients with impaired renal function, MTX dose was recommended to be adjusted to prevent delayed methotrexate elimination and adverse drug reactions of methotrexate [10]. In children with risk factors for methotrexate toxicities, individualized HDMTX doses were proved to be able to avoid methotrexate-related toxicities [11].

In patients with normal renal function, research concerning HDMTX dose was limited and HDMTX was often administered conventionally based on body surface area (BSA). Delayed methotrexate elimination and acute kidney injury (AKI) induced by HDMTX might occur in children with normal renal function, and delayed elimination was reported more likely to occur in the first cycle of HDMTX [12]. Though leukemic infiltration of the kidneys in ALL children is rare, it was reported that severe renal impairment could be a result of leukemic infiltration of the kidneys [13]. It is not clear whether the delayed methotrexate elimination or the occurrence of AKI in the first cycle of HDMTX was caused by the undetected impaired renal function.

Nowadays, renal function was mainly assessed by serum creatinine or serum creatinine-based estimated GFR (eGFR). Researches concerning the accuracy of equations to estimate GFR with serum creatinine in children with malignant diseases found that commonly used equations including Schwartz equation could not reflect the measured GFR perfectly [14, 15]. And in children with ALL, predicted GFRs were poorly correlated with measured GFRs [16]. Serum creatinine was not suitable in assessing GFR of children with ALL.

GFR determined by an isotope clearance method was proved to be accurate in the estimation of GFR in cancer patients [17]. And recently, the GFR measured by ^99m^Tc-DTPA renal dynamic imaging the Gates method (GFR_Gates_) was used to measure GFR and was proved to be well correlated with the two-sample method GFR [18].

In this study, we conducted a prospective investigation to find whether the renal functions of ALL children after induction therapy will affect the occurrence of HDMTX-induced AKI by GFR_Gates_.

## 2. Methods

### 2.1. Patients

Children with ALL between April 2018 and January 2020 receiving HDMTX (dosage over 3 g/m^2^) were included in the study. All the cases consented to receive ^99m^Tc-DTPA renal dynamic imaging and to be enrolled in the study by parents or guardians. The study was approved by the Research Ethics Board of Wuhan Children's Hospital.

Clinical data were recorded from the medical record including age, gender, body weight, height, MTX dosage per BSA, serum creatinine, and alanine aminotransferase (ALT).

### 2.2. Treatment

All children were treated following the China treatment recommendations for ALL (Chinese Children's Leukemia Group–ALL 2008, CCLG–ALL 2008). The detailed treatment of HDMTX has been described in another study [19]. Briefly, children with ALL received different dosages of HDMTX according to their risk categories by a continuous 24-hour intravenous infusion. Children with low-risk ALL received MTX 2 g/m^2^, whereas moderate- and high-risk children received MTX 5 g/m^2^. As patients receiving MTX 2 g/m^2^ were not likely to suffer from HDMTX-induced AKI, patients receiving MTX 2 g/m^2^ were not included in the study. Considering that some doses of MTX might be modulated due to impaired renal functions in moderate- and high-risk children who were expected to receive MTX 5 g/m^2^, patients receiving MTX over 3 g/m^2^ were included in the present study. Hyperhydration and urine alkalinization (pH > 7) were used to enhance the elimination and solubility of methotrexate in the urine. The moderate- and high-risk children receiving dosage over 3 g/m^2^ were included in our study.

eGFR was calculated with the Schwartz equation [20].

Before the first administration of HDMTX, all the cases consented to receive ^99m^Tc-DTPA renal dynamic imaging, and ^99m^Tc-DTPA renal dynamic imaging Gates method (GFR_Gates_) was standardized to the standard BSA of 1.73 m^2^ (standardized GFR_Gates_).

Patients were eligible for HDMTX if they had a normal hepatic function, a white blood cell count ≥ 1.5 × 10^9^/l, a neutrophil cell count ≥ 0.5 × 10^9^/l, a platelet count ≥ 50 × 10^9^/l, and without any evidence of severe infection. The doses of HDMTX were modulated when patients' GFR were below 60 ml/min per 1.73 m^2^ [10].

All children received the same supportive care. The detailed preventive methods of delayed MTX elimination and the administration of leucovorin were described in another study [19].

MTX-induced AKI was defined according to Kidney Disease Improving Global Outcomes classification [21] within 7 days after the initiation of the HDMTX infusion.

For patients with AKI and delayed MTX elimination, hydration, alkalinization, and a prompt increase in the LV dose based on plasma MTX concentrations were used as the main mode of therapy in patients with adequate urine output. Patients with acute kidney failure received transient hemodialysis.

### 2.3. Statistical Analysis

Statistical analysis was performed using the Statistical Package for the Social Sciences (SPSS version 21.0, SPSS Inc., Chicago, IL, USA). By univariate analysis, normally distributed continuous variables were presented as means with standard deviations (SD) and compared with a Student *t*-test. Nonnormally distributed continuous variable data were presented as medians with interquartile range (IQR) and compared with a Mann–Whitney *U* test. Categorical data were compared using a chi-squared test. Variables with a *p* value < 0.1 in univariate analyses were analyzed by a stepwise backward binary logistic regression to identify independent risk factors for the occurrence of MTX-induced AKI. Nonparametric calculation of the area under the curve (AUC) of the receiver-operating characteristic curve (ROC) was used for assessing variables' ability to predict AKI. Variables with a two-tailed *p* value < 0.05 were considered statistically significant.

## 3. Results

102 children with ALL were included in the study. The patients' demographic characteristics (stratified by the occurrence of AKI) are shown in Table 1. Of the 102 children, 21 children developed AKI.

The normal renal function of children was reported to be above 80 ml/min per 1.73 m^2^ [22]. When measured with the ^99m^Tc-DTPA renal dynamic imaging Gates method, the GFR of only one child (74.6 ml/min per 1.73 m^2^) was below 80 ml/min per 1.73 m^2^. As the modulation of MTX dose based on GFR was recommend when the GFR was below 60 ml/min per 1.73 m^2^ [10] and there was no guidance on how to individualize the dose of HDMTX with results of the standardized GFR_Gates_, at this stage of the investigation, the doses of HDMTX were not administered individually according to the value of the standardized GFR_Gates_. The child whose standardized GFR_Gates_ was 74.6 ml/min per 1.73 m^2^ did not suffer from HDMTX-induced AKI.

Univariate analysis showed that there were significant differences in age, body weight, height, standardized GFR_Gates_, MTX24h, MTX48h, and MTX72h. Children with HDMTX-induced AKI were older.

There were no significant differences in gender, risk categories, MTX dose per BSA, serum creatinine, ALT, ALB, hydration on the first day of HDMTX, frequency of urine pH > 7.0 on the first day of HDMTX, and eGFR between the two groups. The serum creatinine levels were higher in the AKI group though it was in a normal range, and the difference was not significant on a 0.05 p level between the AKI group and non-AKI group.

**Figure 1:**
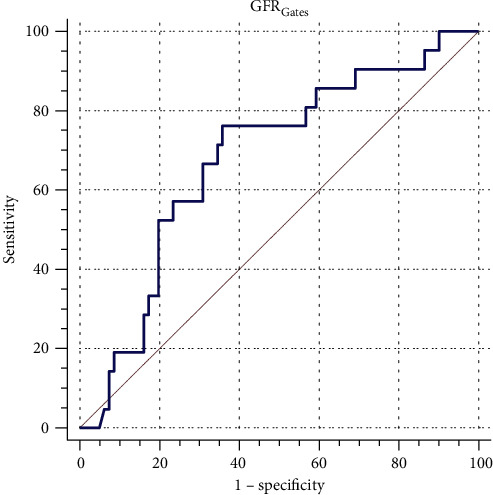
ROC curve of standardized GFR_Gates_. The area under the ROC was 0.679 (*p* = 0.012, 95% CI 0.554-0.804).

Variables (including age, body weight, height, standardized GFR_Gates_, and serum creatinine) with a *p* value < 0.1 in univariate analyses were analyzed by a stepwise backward binary logistic regression. The results showed that only the standardized GFR_Gates_ was an independent risk factor for HDMTX-induced AKI (*p* = 0.018, odds ratio 0.985, 95% CI 0.972-0.997). Then, the standardized GFR_Gates_ was analyzed by the ROC curve to assess its ability to predict HDMTX-induced AKI (Figure 1). The result showed that the area under the ROC was 0.679 (*p* = 0.012, 95% CI 0.554-0.804). The cutoff value of the standardized GFR_Gates_ for the occurrence of HDMTX-induced AKI was 179.61 ml/min per 1.73 m^2^ (the sensitivity and specificity of the cutoff value were 64% and 76%, respectively).

## 4. Discussion

HDMTX therapy is an important chemotherapy in decreasing the systemic relapse rate of ALL. Appropriate methotrexate concentration could improve treatment outcomes and decrease dose-related toxicities. Accurate knowledge of GFR could help oncologists make critical dosage decisions.

A study showed that among the investigated factors, only age and body weight can influence HDMTX clearance [23]. In the present study, univariate analysis showed that children in the AKI group were significantly older and heavier. It seemed that age and body weight also influenced the occurrence of HDMTX-induced AKI. However, logistic regression showed that none of the age, body weight, and serum creatinine had a significant influence on the occurrence of HDMTX-induced AKI. As the serum creatinine level could be influenced by age [24], the older age in the AKI group might be one of the reasons of the higher serum creatinine in the AKI group which indicated that serum creatinine level might not be suitable in modulating HDMTX dose or guiding the supportive care in children with ALL.

In the present study, renal functions were assessed by eGFR and GFR_Gates_. All the values of eGFR and most of the values of standardized GFR_Gates_ were higher than 80 ml/min per 1.73 m^2^ which showed normal renal function after induction therapy and was consistent with research about the renal function of ALL children after induction therapy [25]. However, there was a disparity between the two different methods on whether renal functions before the administration of HDMTX had an influence on the occurrence of HDMTX-induced AKI. Renal function obtained by eGFR showed no significant influence on the occurrence of HDMTX-induced AKI, whereas renal function assessed by the standardized GFR_Gates_ showed significant influence on it. The cutoff value of the standardized GFR_Gates_ was 179.61 ml/min per 1.73 m^2^. It seemed that the normal renal function of children is not enough to be used as a cutoff point to predict the occurrence of AKI.

Logistic regression showed that the standardized GFR_Gates_ was an independent predicting factor of HDMTX-induced AKI; however, the odds ratio was only 0.972 and the area under ROC was only 0.679 which indicated that the ability of the standardized GFR_Gates_ to predict HDMTX-induced AKI was poor. However, as host risk factors including GFR combined with other risk factors can enhance the risk for nephrotoxicity [26], more attention and supportive care should be given to the children with a standardized GFR_Gates_ lower than the cutoff value as they are more likely to develop HDMTX-induced AKI. In ALL children, after induction therapy, measuring GFR by the ^99m^Tc-DTPA renal dynamic imaging Gates method is applicable.

As concurrent use of proton-pump inhibitors (PPI) and nonsteroidal anti-inflammatory drugs (NSAIDs) was not found to be correlated with delayed methotrexate elimination or HDMTX-induced AKI in previous studies [19, 27], in this study, the concomitant use of PPI and NSAIDs was not investigated.

There was one limitation to this study. This was a single-center investigation, and the number of patients enrolled was relatively small. The cutoff value of the standardized GFR_Gates_ for predicting HDMTX-induced AKI in children with ALL should be further investigated.

## 5. Conclusion

The standardized GFR_Gates_ showed that the normal renal function of children is not enough to be used as a cutoff point to predict HDMTX-induced AKI in ALL children receiving HDMTX. More attention and supportive care should be given to the children with a standardized GFR_Gates_ lower than the cutoff value to avoid the HDMTX-induced AKI. The cutoff value of standardized GFR_Gates_ for predicting HDMTX-induced AKI in children with ALL should be further investigated.

## Figures and Tables

**Table 1:** The patients' demographic characteristics.

Characteristic	Non-AKI	AKI	*p* value
Number of patients (*n*)	81	21	—
Age (years) (median (IQR))	4.70 (3.08-7.83)	7.75 (5.17-11.17)	0.016^b^
Gender (male/female) (*n*)	47/34	15/6	0.262^c^
Body weight (kg) (median(IQR))	17.50 (14.00-23.50)	26.00 (18.25-30.00)	0.008^b^
Height (cm) (mean (SD))	111.75 (23.55)	124.62 (22.32)	0.026^a^
Moderate risk/high risk (*n*)	47/34	12/9	0.942^c^
eGFR (ml/min per 1.73 m^2^) (mean (SD))^∗^	226.39 (51.58)	222.76 (43.29)	0.768^a^
Standardized GFR_Gates_ (ml/min per 1.73 m^2^) (mean (SD))	215.59 (79.76)	168.10 (67.13)	0.014^a^
Dosage/m^2^ (g) (median (IQR))	4.92 (4.25-5.14)	4.75 (4.35-4.98)	0.331^b^
Serum creatinine (*μ*mol/l) (median (IQR))	22.60 (19.15-31.40)	26.80 (22.55-32.90)	0.085^b^
ALT (U/l) (median (IQR))	16.0 (11.0-27.0)	17.0 (11.5-37.0)	0.469^b^
ALB (g/l) (mean (SD))	45.34 (4.57)	45.08 (4.19)	0.816^a^
Hydration on the first day of HDMTX (ml/m^2^) (mean (SD))	3392 (597)	3562 (751)	0.274^a^
Patients with urine pH > 7.0 on the first day of HDMTX, *n* (%)	76 (93.8)	18 (85.7)	0.437^c^
MTX24h (*μ*mol/l) (mean (SD))	58.02 (21.42)	81.90 (30.34)	≤0.001^a^
MTX48h (*μ*mol/l) (median (IQR))	0.49 (0.35-0.86)	5.21 (3.37-19.65)	≤0.001^b^
MTX72h (*μ*mol/l) (median (IQR))	0.11 (0.07-0.18)	1.94 (0.76-6.35)	≤0.001^b^

^a^Normally distributed continuous variables were reported as means with standard deviations (SD) and compared with Student's *t*-test. ^b^Nonnormally distributed continuous variable data were reported as medians with interquartile range (IQR) and compared with Mann–Whitney *U* test. ^c^Categorical data were reported as proportions (*n* with %) and compared using chi-squared test. ^∗^eGFR was calculated with the Schwartz equation.

## Data Availability

The data used to support the findings of this study are available from the corresponding author through polarisyxt@hotmail.com on request.

## Abbreviations

AKI: Acute kidney injury
ALL: Acute lymphoblastic leukemia
ALT: Alanine aminotransferase
AUC: Area under the curve
BSA: Body surface area
Ccr: Creatinine clearance rate
eGFR: Estimated GFR
GFR: Glomerular filtration rate
GFR_Gates_: GFR measured by ^99m^Tc-DTPA renal dynamic imaging Gates method
HDMTX: High-dose methotrexate
IQR: Interquartile range
NSAIDs: Nonsteroidal anti-inflammatory drugs
PPI: Proton-pump inhibitors
ROC: Receiver-operating characteristic curve
SD: Standard deviations
Standardized GFR_Gates_: GFR_Gates_ standardized to the standard BSA of 1.73 m^2^.
